# Associations of multiple serum biomarkers and the risk of cardiovascular disease in China

**DOI:** 10.1186/s12872-020-01696-7

**Published:** 2020-09-29

**Authors:** Huichen Yao, Chenyang Hou, Weihua Liu, Jihu Yi, Wencong Su, Qingzhi Hou

**Affiliations:** 1 The third affiliated hospital of Shandong first medical university, Jinan, Shandong China; 2 Guangxi Medical University, Nanning, Guangxi China; 3 Shandong First Medical University (Shandong Academy of Medical Sciences), 169 Great Wall Rd, Taian, 271000 Shandong China; 4 Zibo Bashan Wanjie Hospital, Zibo, Shandong China

**Keywords:** Serum, Biomarkers, Cardiovascular disease, Multiple, Relationship

## Abstract

**Background:**

Previous studies focus on one or several serum biomarkers and the risk of cardiovascular disease (CVD). This study aims to investigate the association of multiple serum biomarkers and the risk of CVD and evaluate the dose-relationship between a single serum metabolite and CVD.

**Methods:**

Our case-control study included 161 CVD and 160 non-CVD patients who had a physical examination in the same hospital. We used stratified analysis and cubic restricted analysis to investigate the dose-response relationship of individual serum biomarkers and the CVD incident. Moreover, to investigate serum biomarkers and CVD, we used elastic net regression and logistic regression to build a multi-biomarker model.

**Results:**

In a single serum biomarker model, we found serum FT4, T4. GLU, CREA, TG and LDL-c were positively associated with CVD. In the male group, serum T4, GLU and LDL-c were positively associated with CVD; and serum TG was positively associated with CVD in the female group. When patients ≤63 years old, serum T4, GLU, CREA and TG were positively associated with CVD, and serum TG and LDL-c were positively associated with CVD when patients > 63 years old. Moreover, serum GLU had nonlinearity relationship with CVD and serum TG and LDL-c had linearity association with CVD. Furthermore, we used elastic regression selecting 5 serum biomarkers (GLU, FT4, TG, HDL-c, LDL-c) which were independently associated with CVD incident and built multi-biomarker model. And the multi-biomarker model had much better sensitivity than single biomarker model.

**Conclusion:**

The multi-biomarker model had much higher sensitivity than a single biomarker model for the prediction of CVD. Serum FT4, TG and LDL-c were positively associated with the risk of CVD in single and multiple serum biomarkers models, and serum TG and LDL-c had linearity relationship with CVD.

## Background

Cardiovascular disease (CVD) refers to the coronary artery atherosclerotic lesions that cause arteries to narrow. Myocardial ischemia caused by lack of oxygen in heart disease is the major threat for human health in China. The morbidity and mortality of CVD are increasing in China [1].

Smoking, drinking, hypertension and diabetes are relative to the risk of CVD [2]. Recent studies reported that serum biomarkers such as blood glucose (GLU), high-density lipoprotein cholesterol (HDL-c), low-density lipoprotein cholesterol (LDL-c), Uric Acid (UA), thyroid-stimulating hormone (TSH), Homocysteine (Hcy) and other biomarkers were associated with the risk of CVD [2–8]. However, the results were inconsistent, and most of them focused only on one or several biomarkers, neglected the effect of multi-biomarker for the risk of CVD.

Based on our case-control study, we aimed to investigate the associations between single and multiple serum biomarkers and the risk of CVD and to investigate the dose-response relationship of a single serum biomarker and the risk of CVD.

## Methods

### Study design and population

Our study was approved by the Medical Ethics Committee of Zibo Bashan Wanjie Hospital, the number is 2019 (03). All participants were our hospital patients, in later follow-up, they consented to participate in this research and offered written informed consent.

In the case-control study, patients who had chest pain and underwent coronary angiography at Da Yi Hu Cardiovascular Hospital from November 2014 to October 2017 were included. Patients with a history of coronary artery disease and the history of heart operation were excluded. Controls were randomly selected from patients who had average physical examination results in this hospital at the same time. The inclusion and exclusion criteria and case-control matched 1:1, and 321 patients were included with 161 CVD and 160 non-CVD patients.

Serum biomarkers included in our study were measured in our hospital laboratory. Thyroxine levels including TSH, T3, T4, FT3 and FT4 were measured by radioimmunoassay (Roche). Moreover, we measured blood lipids levels including HDL-c, LDL-c, triglycerides (TG) and total cholesterol (TC) by electrochemical luminescence (OLYMPUS), and CREA, UA and Hcy were measured by the biochemical analyser (Advia2400). At last, fasting blood glucose was determined by the glucose oxidase assay.

Several types of data were collected from the medical records, including questions about prior use of alcohol and tobacco, medical history, family history of diabetes and hypertension, as well as other demographic and socioeconomic information (weight and height, age, sex, etc.), BMI was calculated as weight (kg) divided by square meters of height (m^2^).

### Definition of CVD

Coronary angiography is the golden standard for the diagnosis of CVD. Left main artery (LM), left anterior descending artery (LAD), cyclotron artery (LCX) and right coronary artery (RCA) with stenosis over 50% defined CVD [9]. For coronary angiography, we used GE digital plate angiography system (INNOVA 4100 IQ).

### Statistical analyses

We performed a descriptive analysis to describe the baseline characteristics of CVD and non-CVD. T-test and Kruskal Wallis H test were used to compare the continuous variables, and chi-square tests were used to compare the categorical variables during two groups. We used Spearman’s rank correlation analysis tocalculate the correlations of concentration for 13 serum biomarkers.

To investigate the association between multiple serum biomarkers concentrations and the risk of CVD, we built a single and multi-biomarker model. In the single biomarker model, all serum biomarker concentrations were divided into tertiles as classification variables. It contained unadjusted and adjusted models, and the adjusted factors were age, gender, smoking, drinking and the history of hypertension and diabetes. To further explore the association between serum biomarker and the risk of CVD for different patients, subgroup analyses were done by median age (≤63 and>63 years old) and gender (male and female). Furthermore, we used restricted cubic splines regression (RSC) to explore the dose-response relationship of a single serum biomarker and the risk of CVD [10].

To further investigate the multi-biomarker model and the risk of CVD, we used elastic net regression [11], which could improve prediction accuracy and model interpretation by decreasing over-fitting to select serum biomarkers independently associated with CVD. And then webuilt a multi-biomarker model by logistic regression, which contained unadjusted and adjusted models, and the adjusted factors were the same as the single biomarker model.

At last, to compare the prediction sensitivity of the single biomarker and the multi-biomarker models and the risk of CVD, we made ROC curve analyses of these two models and compared the difference by Ztest.

We used R version 3.4.2 (64 bit) and SAS (version 9.4; SAS Institute Inc.) to perform all the analyses. A *P* value of two sides less than 0.05 was considered as statistically significant.

## Results

### Characteristics of the Study population

Table 1 showed the characteristics of 321 eligible participants at baseline stage, 161 CVD and 160 non-CVD. Compared to the non-CVD group, CVD participants had a higher prevalence of a history of smoking, drinking, hypertension, diabetes mellitus and present hypertension (all *P* <  0.05). Moreover, the gender, age(≤60and > 60 years old) and systolic pressure had statistical difference during two groups (all *P* <  0.05). In contrast, BMI (kg/m^2^) and diastolic pressure had no statistical difference for CVD and non-CVD (all *P* > 0.05).

**Table 1:** The baseline characters of the CVD and non-CVD

	CVD	non-CVD	*P*
n	161	160	
Age (Years old)	62.73 ± 7.16	59.24 ± 7.83	< 0.001
≤ 60	51 (31.7)	86 (53.8)	
> 60	110 (68.3)	74 (46.2)	
Gender			0.004
Male	91 (56.5)	65 (40.6)	
Female	70 (43.5)	95 (59.4)	
BMI (kg/m^2^)			0.056
≤ 18.5	41 (34.8)	40 (22.3)	
18.6–23.9	80 (54.9)	79 (68.6)	
≥ 24	40 (10.2)	41 (9.1)	
History of smoking			0.022
Yes	37 (23.0)	21 (13.1)	
No	124 (77.0)	139 (86.9)	
History of drinking			0.008
Yes	36 (22.4)	18 (11.2)	
No	125 (77.6)	142 (88.8)	
History of hypertension			0.024
Yes	114 (70.8)	94 (48.7)	
No	47 (29.2)	66 (41.3)	
History of diabetes			0.001
Yes	53 (32.9)	27 (16.9)	
No	108 (67.1)	133 (83.2)	
Hypertension			0.048
Yes	104 (64.6)	86 (53.7)	
No	57 (35.4)	74 (46.3)	
Systolic pressure^a^	148.48 ± 22.84	142.43 ± 22.24	0.017
Diastolic pressure^a^	85.40 ± 11.16	84.05 ± 11.89	0.231

^a^t-test

### Levels of serum biomarkers

Table 2 summarised the serum chemical biomarkers concentrations. Except for serum GLU, TG, LDL-c and HDL had statistic difference for CVD and non-CVD groups (all *P* value < 0.05), other serum biomarkers concentrations had no statistical difference (all *P* value > 0.05). The Spearman’s rank correlation coefficients showed that some serum biomarkers were significantly correlated with one another (*P* values < 0.05, r = − 0.530 to 0.834, Fig. S1).

**Table 2:** The serum levels of biomarkers

biomarkers		n	Min	25th	50th	75th	Max	*P*
TSH (mIU/L)								0.734
	Case	161	0.16	1.36	1.98	2.83	21.99	
	Control	160	0.06	1.39	1.95	2.84	12.24	
FT3 (pmol/L)								0.153
	Case	161	2.27	4.51	4.99	5.46	7.04	
	Control	160	2.03	4.40	4.79	5.38	7.40	
FT4 (pmol/L)								0.032
	Case	161	6.30	15.99	17.92	19.43	23.72	
	Control	160	10.40	15.01	17.14	19.13	25.02	
T3 (nmol/L)								0.976
	Case	161	1.34	1.69	1.98	2.25	3.28	
	Control	160	1.28	1.76	1.98	2.20	2.96	
T4 (nmol/L)								0.299
	Case	161	49.08	90.66	105.50	118.50	165.30	
	Control	160	54.28	86.13	101.45	118.38	168.70	
GLU (mmol/L)								< 0.001
	Case	161	1.11	4.95	5.66	6.95	16.46	
	Control	160	3.95	4.74	5.10	5.75	14.31	
CREA (μ mol/L)								0.299
	Case	161	35.00	51.00	61.00	71.00	160.00	
	Control	160	32.00	52.00	60.50	69.00	621.00	
UA (μ mol/L)								0.061
	Case	161	45.00	235.50	276.00	328.50	573.00	
	Control	160	113.00	217.00	265.00	312.00	638.00	
TC (mmol/L)								0.214
	Case	161	2.31	4.27	4.98	5.57	7.67	
	Control	160	2.59	4.18	4.72	5.49	8.18	
TG (mmol/L)								< 0.001
	Case	161	0.54	1.15	1.61	2.19	9.14	
	Control	160	0.34	0.87	1.27	1.72	7.76	
HDL-c (mmol/L)								0.018
	Case	161	0.50	0.92	1.06	1.26	2.13	
	Control	160	0.50	0.98	1.11	1.32	1.91	
LDL-c (mmol/L)								0.013
	Case	161	1.32	2.48	2.89	3.41	6.44	
	Control	160	0.97	2.30	2.70	3.15	5.23	
HCY (μ mol/L)								0.693
	Case	161	5.50	9.35	11.10	13.50	44.50	
	Control	160	4.60	9.60	11.20	13.78	60.70	

### A single serum biomarker and the risk of CVD

**Fig. 1:**
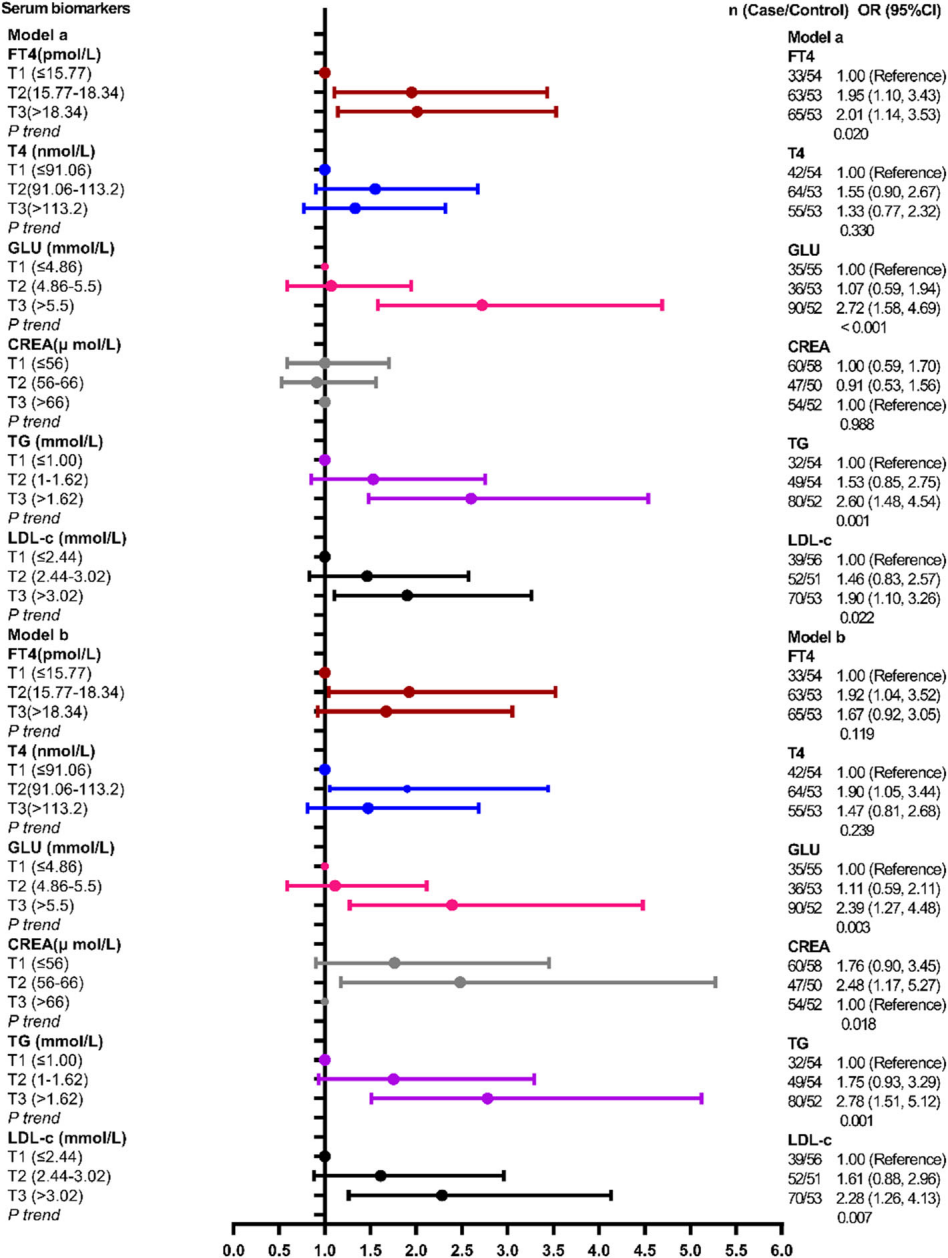
The logistic regression analysis of single serum biomarkers and the risk of CVD. Model a was unadjusted model and Model b was adjusted model and the adjusted factors were age (≤ 60 and > 60 years old), gender, the history of hypertension, diabetes, smoking and drinking

In the single biomarker model, serum biomarkers concentrations were divided into tertiles as classification variables, and the adjusted factors were age (≤60 and > 60 years old), gender, smoking, drinking and the history of hypertension and diabetes. Except serumCREA set T3 concentration as a reference, other serum biomarkers were all set T1 concentration as reference. Serum FT4, GLU, TG and LDL-c had statistical associations with the risk of CVD in unadjusted and adjusted regression analysis models (FT4: Unadjusted model: T2 vs T1, Unadjusted OR = 1.95, 95%CI:1.10–3.43; T3 vs T1: Unadjusted OR = 2.01, 95%CI: 1.14–3.53, *P* trend = 0.020. Adjusted model: T2 vs T1: Adjusted OR = 1.92, 95%CI:1.04–3.52, *P* trend = 0.119. GLU: T3 vs T1: Unadjusted OR = 2.72, 95%CI: 1.58–4.69, *P* trend < 0.001; Adjusted OR = 2.39, 95%CI: 1.27–4.48, *P* trend = 0.003. TG: T3 vs T1: Unadjusted OR = 2.60, 95%CI:1.48–4.54, *P* trend = 0.001; Adjusted OR = 2.78, 95%CI: 1.51–5.12) *P* trend = 0.001.LDL-c: T3*vs*T1: Unadjusted OR = 1.90, 95%CI:1.10–3.26, *P* trend = 0.022; Adjusted OR = 2.28, 95%CI:1.26–4.13, *P* trend = 0.007). Moreover, serum T4 and CREA had statistical associations with CVD incident in the adjusted regression model (T4: Adjusted OR was 1.90 (95%CI:1.05, 3.44) for T2 vs T1, *P* trend = 0.239; CREA:Adjusted OR was 2.48 (95%CI: 1.17, 5.27) for T2 vs T3, *P* trend = 0.018),and had no statistical association with CVD incident in the unadjusted logistic regression model (Fig. 1). Other serum biomarkers which had no statistical associations with the risk of CVD were showing in Table S1.

### Subgroup analysis of a single serum biomarker and the risk of CVD

**Fig. 2:**
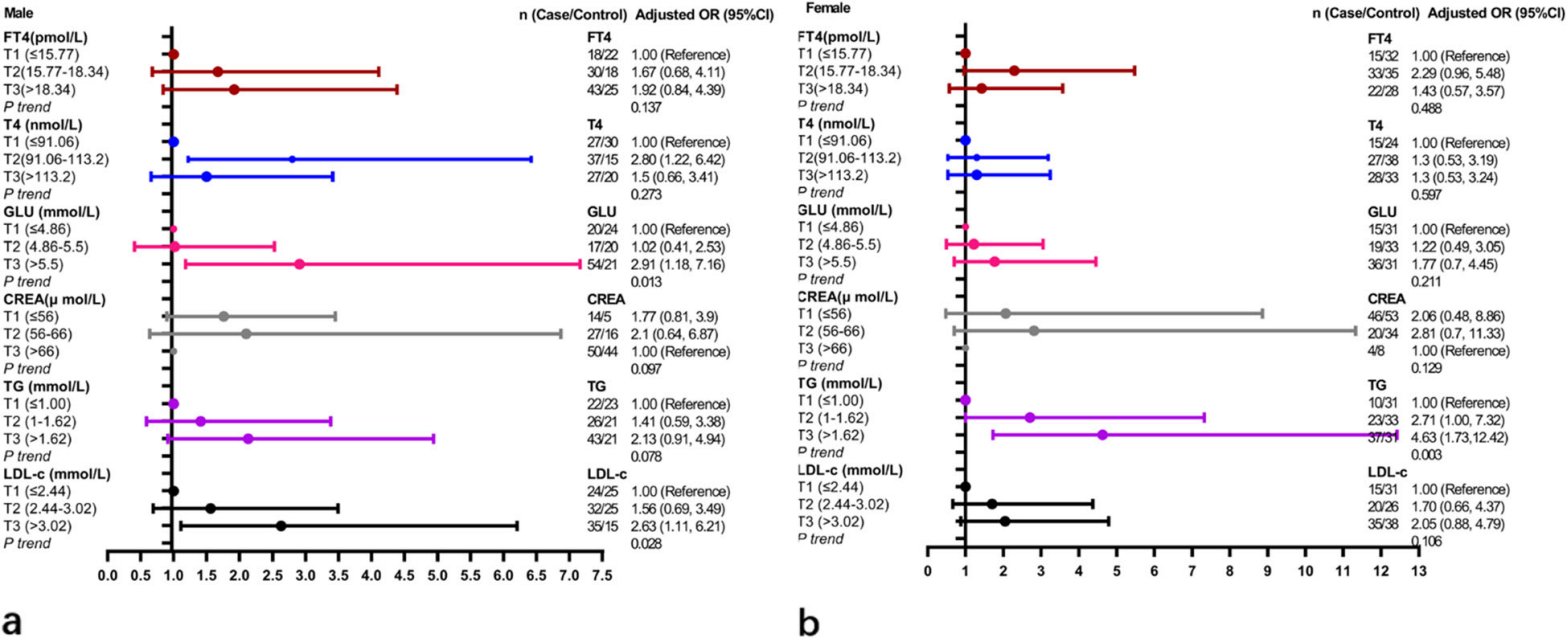
The logistic regression analysis of serum biomarker and CVD incident satisfied by gender. **a** Male; **b** Female. Model a was unadjusted model and Model b was adjusted model and the adjusted factors were age (≤ 60 and > 60 years old), the history of hypertension, diabetes, smoking and drinking

Biomarkers containing FT4, T4, GLU, CREA, TG and LDL-c, which had statistical associations with the risk of CVD in adjusted logistic regression model were included in the subgroup analysis. When subgroup by gender, the adjusted factors of the adjusted logistic regression model were age (≤60 and > 60 years old), the history of hypertension, diabetes, smoking and drinking. When the patient was man, the results showed that higher level serum T4 and GLU were positively associated with the risk of CVD (T4: T2 vs T1: Unadjusted OR = 2.74, 95%CI: 1.24–6.06, *P* trend = 0.248; Adjusted OR = 2.80, 95% CI: 1.22–6.42, *P* trend = 0.273. GLU: T3 vs T1: Unadjusted OR = 3.09, 95%CI: 1.42–6.72, *P* trend = 0.001; Adjusted OR = 2.91, 95% CI: 1.18–7.16 for T3 vs T1, *P* trend = 0.013). Serum LDL-c were positively associated with the risk of CVD in the adjusted model (T3 vs T1: Adjusted OR = 2.63, 95% CI: 1.11–6.21, *P* trend = 0.028) and had no statistical association with CVD incident in unadjusted logistic regression model (*P* and *P* trend values were all > 0.05). Serum FT4, CREA and TG had no statistical association with the risk of CVD in unadjusted and adjusted models, all *P* value > 0.05 (Fig. 2a and Table S2). When the patient was women, higher-level serum TG was positively associated with the CVD incident in unadjusted and adjusted logistic regression models (T3 vs T1: Unadjusted OR = 3.70, 95%CI: 1.57–8.72, *P* trend = 0.003. Adjusted OR = 4.63, 95% CI: 1.73–12.42, *P* trend = 0.003) and serum GLU was positively associated with CVD only in unadjusted logistic model (T3 vs T1: Unadjusted OR = 2.40, 95%CI: 1.10–8.72, *P* trend = 0.016), and other serum chemical biomarkers had no statistical association with the risk of CVD (all *P* value > 0.05) (Fig. 2b and Table S2).

**Fig. 3:**
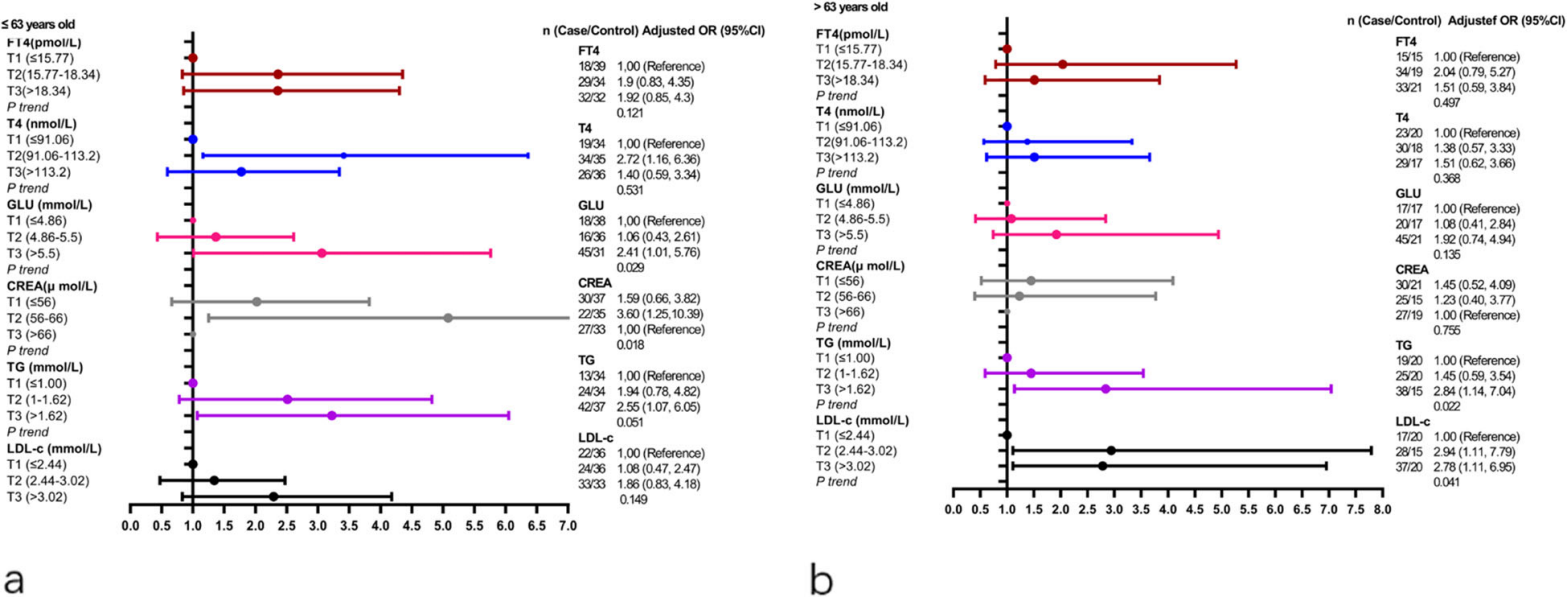
The logistic regression analysis of serum biomarker and CVD incident satisfied by age. **a** ≤ 63 years old; **b** > 63 years old. Model a was unadjusted model and Model b was adjusted model and the adjusted factors were gender, the history of hypertension, diabetes, smoking and drinking

When subgroup by age, the adjusted factors of the adjusted logistic regression model were gender, the history of hypertension, diabetes, smoking and drinking. When patient age ≤ 63 years old, higher levels of serum GLU and TG were positively associated with the risk of CVD (GLU: T3 vs T1: Unadjusted OR = 3.07, 95% CI: 1.49–6.32, *P* trend < 0.001; Adjusted OR = 2.41, 95% CI:1.01–5.76, *P* trend =0.029. TG: T3 vs T1:Unadjusted OR = 2.97, 95% CI: 1.37–6.46, *P* trend = 0.006; Adjusted OR = 2.55, 95%CI: 1.07–6.05, *P* trend = 0.051);Serum T4 and CREA were positively associated with the CVD incident in adjusted logistic regression model(T4: T2*vs*T1: Adjusted OR = 2.72, 95%CI:1.16–6.36, *P* trend = 0.531; CREA: T2*vs*T3: Adjusted OR = 3.60, 95%CI:1.25–10.39, *P* trend = 0.018), and serum FT4 and LDL-c were not associated with CVD incident in unadjusted and adjusted logistic regression models (all *P* and *P* trend values > 0.05) (Fig. 3a and Table S3). For patients> 63 years old, higher level serum TG was positively associated with the risk of CVD (TG: T3 vs T1: Unadjusted OR = 2.67, 95% CI: 1.12–6.35, *P* trend = 0.021; Adjusted OR = 2.84, 95% CI: 1.14–7.04,*P* trend = 0.022), higher-level serum LDL-c was positively associated with the risk of CVD only in adjusted logistic model (T2 vs T1: Adjusted OR = 2.94, 95% CI: 1.11–7.99; T3 vs T1: Adjusted OR = 2.78, 95% CI: 1.11–6.95,*P* trend = 0.041) and had no statistic association with CVD in unadjusted model (all *P* value > 0.05). Furthermore, other serum chemical biomarkers were not associated with the CVD incident in unadjusted and adjusted models (all *P* > 0.05) (Fig. 3 b and Table S3).

To further explore the potential dose-response associations between serum chemical biomarkers and the risk of CVD, we used cubic spline regression models. The biomarkers which had a statistical association with CVD risk in a single biomarker model were selected for RCS analysis, including FT4, T4, GLU, CREA, TG and LDL-c.The adjustment factors of RCS analysis for each biomarker were age(≤ 60and > 60 years old), the history of hypertension, diabetes, smoking and drinking. Knots of RCS were placed at the 25th, 50th, and 75th percentiles of the serum biomarkers distribution (FT4: 15.595, 17.55, 19.195 pmol/L; T4: 88.075, 103.9, 118.45 nmol/L; GLU: 4.81, 5.30, 6.20 mmol/L; CREA: 52, 61, 70 μmol/L; TG: 0.97, 1.42, 1.97 mmol/L; LDL-c: 2.38, 2.81, 3.29 mmol/L), and the reference value was set at the 50th percentile.

**Fig. 4:**
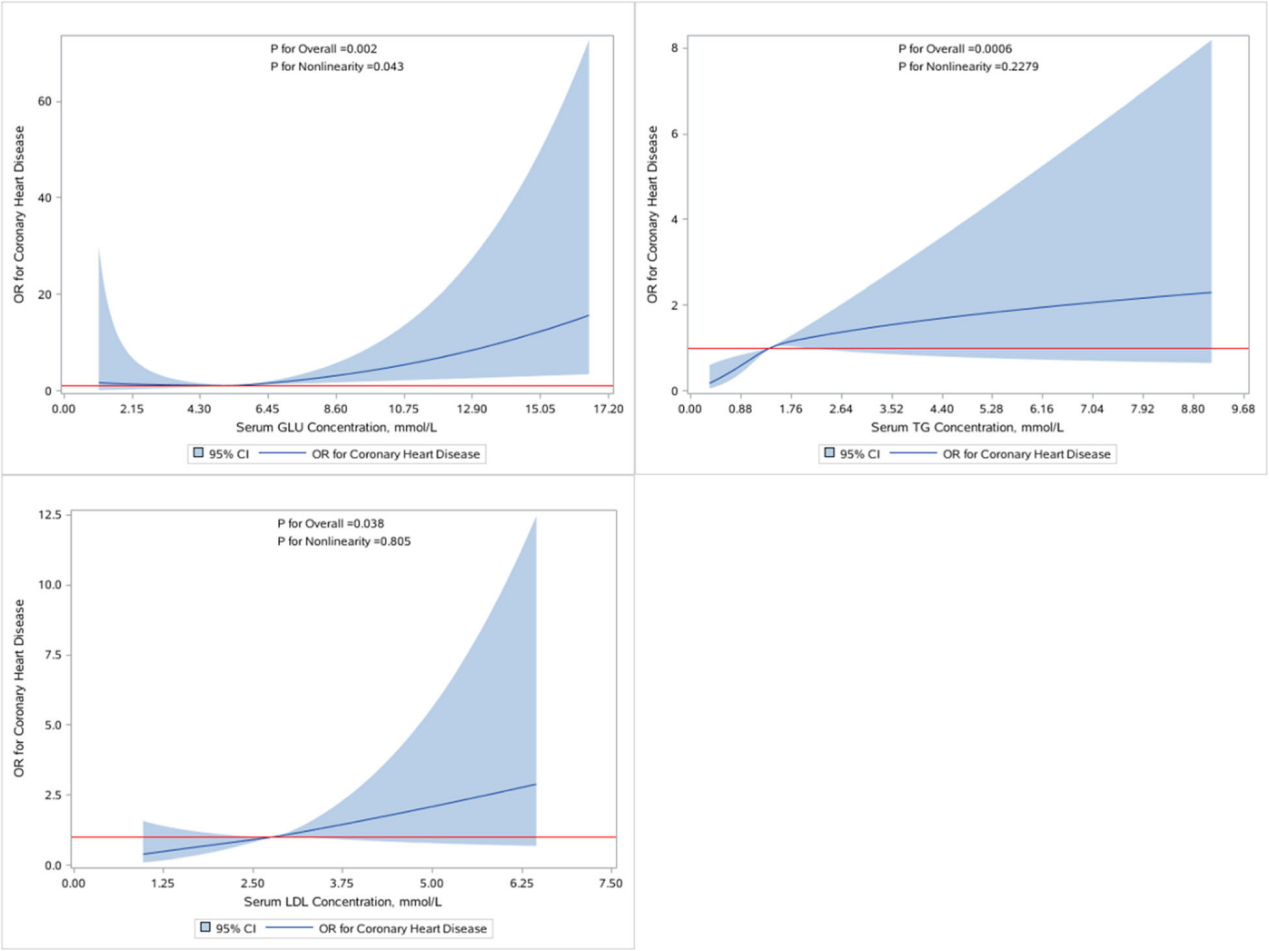
The restricted cubic spline for the association between serum biomarkers and CVD. The lines represent adjusted odds ratios based on restricted cubic splines for the serum biomarker in the multiple-metals conditional regression model. Knots were placed at the 25th, 50th, and 75th percentiles of the serum biomarker distribution, and the reference value was set at the 50th percentile. Adjusted factors were age (≤ 60 and > 60 years old), the history of hypertension, diabetes, smoking and drinking

Interestingly, we found significant non-linear association between blood GLU and CVD (*P*-overall = 0.002, *P*-nonlinearity = 0.043). When blood GLU > 5.30 mmol/L, the risk of CVD was increasing. Meanwhile, we found a linear association between serum TG and LDL-c and the risk of CVD (TG:*P*-overall = 0.001, *P*- nonlinearity = 0.228; LDL-c: *P*-overall = 0.036, *P*- nonlinearity = 0.805), when serum TG and LDL-c concentrations are increasing, the risk of CVD is increasing (Fig. 4). However,FT4, T4 and CREA had no dose-relationship with the risk of CVD **(**Fig. S2**).**

### Multiple serum biomarkers and the risk of CVD

**Fig. 5:**
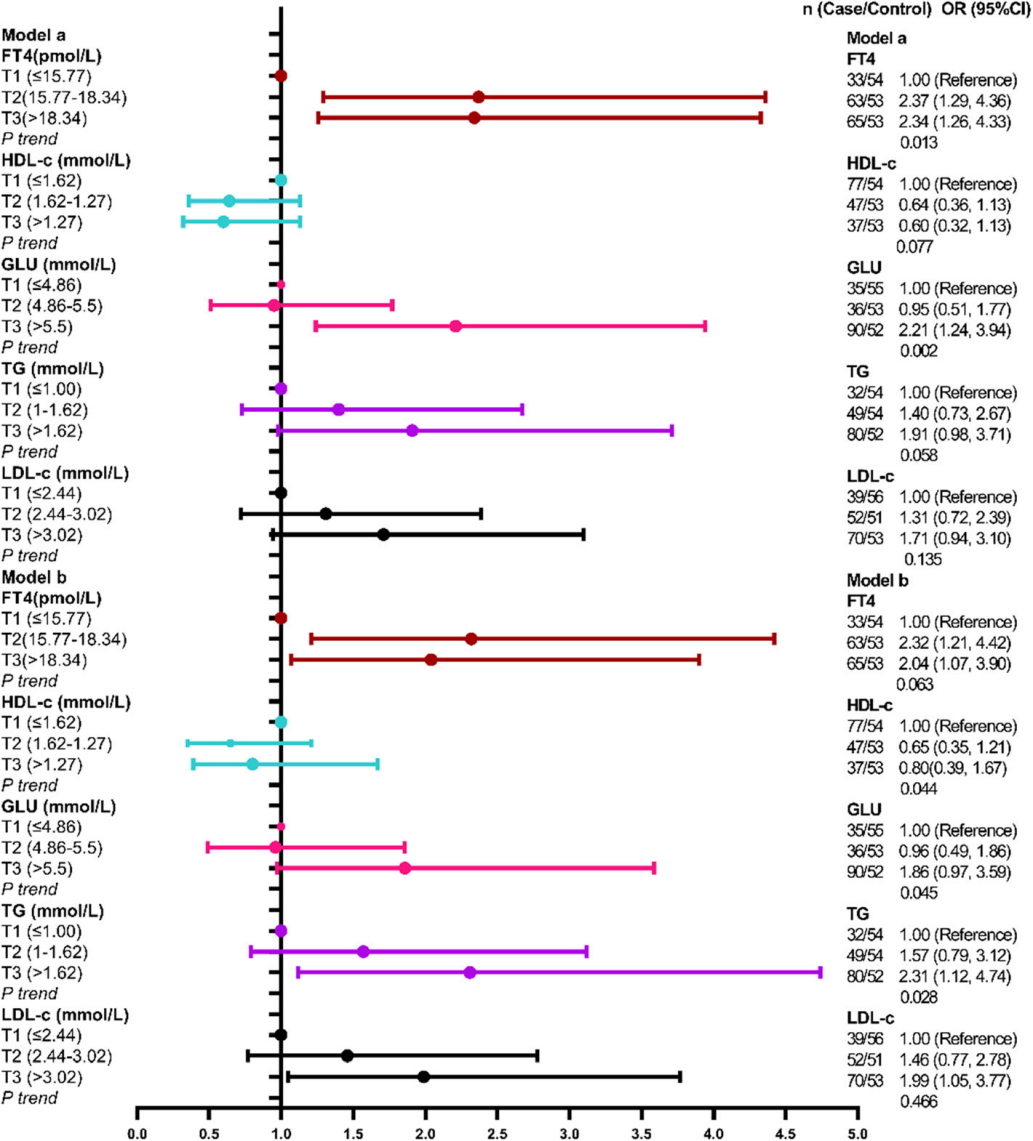
The association of multi-chemical biomarkers selected by elastic net regression and CVD incident. Model a was unadjusted model and Model b was adjusted model and the adjusted factors were age (≤ 60 and > 60 years old), gender, the history of hypertension, diabetes, smoking and drinking

To further investigate the association of multi-serum biomarkers and the risk of CVD, we used elastic regression and logistic regression to build a multi-chemical biomarker model. Because some serum chemical biomarkers had a statistical correlation with others (*P* < 0.05, r = − 0.530 to 0.834), we used elastic net regression to select chemical biomarkers independently associated with the risk of CVD. We selected five chemical metabolites (FT4, TG, GLU, HLD-c, LDL-c) which were associated with the risk of CVD **(**Fig. S3**).** The coefficients of selected biomarkers were 0.019, 0.112, 0.063, − 0.224 and 0.177 which were showing in Table S4.When we analysed the association of multi-chemical biomarkers and the risk of CVD by multi-biomarker logistic regression, the adjusted factors were the same as the single biomarker model. Figure 5 showed that in the unadjusted logistic model, serum FT4 and GLU were positively associated with the risk of CVD (FT4: T2 vs T1: unadjusted OR = 2.37, 95%CI: 1.29–4.36; T3 vs T1: unadjusted OR = 2.34, 95%CI: 1.26–4.33; *P* trend = 0.013. GLU: T2 vs T1: unadjusted OR = 0.95, 95%CI: 0.51–1.77; T3 vs T1: unadjusted OR = 2.21, 95%CI: 1.24–3.94; *P* trend = 0.002). Moreover, in adjusted logistic model, serum FT4, TG and LDL-c were positively associated with the risk of CVD (FT4: T2 vs T1: adjusted OR = 2.32, 95% CI: 1.21–4.42; T3 vs T1: adjusted OR = 2.04, 95% CI: 1.07–3.90; *P* trend = 0.063; TG: T3 vs T1: adjusted OR = 2.31 95% CI: 1.12–4.74; *P* trend = 0.028; LDL-c: T3 vs T1: adjusted OR = 1.99, 95% CI: 1.05–3.77, *P* trend = 0.466).

At last, we made ROC curve analyses of the single biomarker and the multi-biomarker models and the risk of CVD (Fig. S4). The AUCs (95%CI) for serum FT4, GLU, TG and LDL-c in the single biomarker model were 0.569 (0.506, 0.632), 0.636 (0.575, 0.697), 0.628 (0.568, 0.689) and 0.580 (0.518, 0.642); and was 0.660 (0.601, 0.720) in the multi-metabolites model. Z test of AUCs for two models showed that multi-biomarker model had much higher sensitivity than single biomarker of FT4, the *P*_AUC difference_ was 0.038). However, there were no statistical differences between serum GLU, TG, LDL-cand multi-biomarker model; the *P*_AUC difference_ was 0.578, 0.458 and 0.068, respectively **(**Table S5**).**

## Discussion

To explore the relationship between multiple serum biomarkers and CVD incident, we built a single biomarker and a multi-biomarker model and compared the sensitivity of the two models. Our results showed that serum FT4, T4, GLU, CREA, TG and LDL-c were associated with the risk of CVD in the single metabolites model. The relationship was different for different age (≤ 63 years old and > 63 years old) and gender (man and woman), especially for serum GLU, TG and LDL-c. Moreover, serum GLU had nonlinearity relationship with the risk of CVD, when serum GLU > 5.30 mmol/L, the risk of CVD was increasing. Interestingly, we also found serum TG and LDL-c had linearity relationship with the risk of CVD. And then, in the multi-biomarker panel model, serum FT4, TG and LDL-c were positively associated with the risk of CVD.

Previous studies have shown that patients with higher-level GLU, especially with T2DM, more than 75% of patients die of cardiovascular-related diseases, which is two times more than the number in non-diabetic patients [12]. Moreover, reported studies also found that the cardiovascular event group had higher fasting blood glucose and the 2 hours postprandial blood glucose levels than the non-cardiovascular events group [13]. Our study also found that serum GLU was positively associated with the risk of CVD. Interestingly, we also found that serum GLU had nonlinearity dose relationship with the risk of CVD, when serum GLU > 5.30 mmol/L, the risk of CVD was increased. The explanation for this was that long-term higher level GLU could damage the vascular endothelial cells, promoting the migration and proliferation of vascular smooth muscle cells [14]and activationof plasminogen activator inhibitor-1 [15]. At the same time, the procoagulant factors and antithrombotic factors could be increasing, which could promote the endothelial dysfunction [16]and accelerate the formation of thrombosis and the incidence of cardiovascular events.

Furthermore, we also found that higher level of serum TG and LDL-c were positively associated with the risk of CVD, and had linearity dose-relationship with the CVD incident, soan increase of TG and LDL increased the risk of CVD. Abnormal lipid metabolism is the most critical risk factor in atherosclerosis [17]. Previous studies reported that the level of TG and LDL-c were associated with the risk of CVD [17]. Sonmezet al. demonstrated that an elevated TG/HDL-c ratio predicted poor CVD outcome in subjects with chronic kidney diseases (CKD), and proposed this ratio as a simple, inexpensive and reproducible marker of CVD risk in chronic renal diseases [18]. Džubur*et al.* found that the TG/HDL-c ratio was a useful and straight forward biomarker to predict the risk of acute cardiovascular syndrome (ACS) [6]. The explanation for these results was that lipid disorders could lead to atherosclerotic changes, which could accelerate the development of heart disease.

We also found that serum creatinine was associated with CVD incident. We divided the serum creatinine concentration into tertiles, and in an adjusted logistic regression model with the T3 as a reference, only T2 (56–66) μmol/L was associated with CVD incident (Adjusted OR = 2.48, 95%CI:1.17–5.27). Pandya *et al.* found that serum creatinine was associated with CKD [19]. Moreover, serum creatinine was a marker of kidney function, although the mechanism by which an increase in serum creatinine caused CKD is unknown [17]. Previous studies reported that kidney function was associated with the risk of CVD [18, 20].

Interestingly, we also found that the associations of serum GLU, TG and LDL-c and the risk of CVD were different for different sex and age of patients (≤63 years old and > 63 years old). In subgroup analysis, when the patient was male, serum GLU was positively associated with the risk of CVD (Adjusted OR = 2.91, 95%CI: 1.18–7.16. *P* trend = 0.013); when patients ≤63 years old, serum GLU was also positively associated with CVD (Adjusted OR = 2.41, 95%CI: 1.01–5.76, *P* trend = 0.029). Furthermore, when the patient was male and > 63 years old, higher level serum LDL-c was positively associated with the risk of CVD (Male: Adjusted OR = 2.63, 95%CI: 1.11–6.21, *P* trend = 0.028; > 63 years old: T2 vs T1: Adjusted OR = 2.94, 95%CI: 1.11–7.79; T3 vs T1: Adjusted OR = 2.78, 95%CI: 1.11–6.95, *P* trend = 0.041). When the patient was female, higher-level serum TG was positively associated with the risk of CVD (Adjusted OR = 4.63, 95%CI: 1.73–12.42, *P* trend = 0.003). Our results were in accordance with previous studies which found that the concentrations of some serum biomarkers were different for differentage [21]and sex [22]. Of course, we should further verify them in other populations and animal experiments.

Moreover, the multi-biomarker model had much higher sensitivity than the single biomarker model. Very few studies examined the association between serum metabolomics and the risk of CVD. We used elastic net regression and logistic regression to build the multi-serum biomarker model. Elastic net regression could select independent biomarkers associated with the risk of CVD. Moreover, the multi-biomarker model could systematically measure the associations between metabolomics and the risk of CVD. Our findings provided new insight into the future investigation of the association between multiple biomarkers and the risk of CVD.

An important strength of our study was the building of the multi-biomarker model completed by elastic net regression and logistic regression. Since human beings are often co-exposed to multiple biomarkers rather than a single biomarker, we select variables and construct the multi-biomarker model by elastic net regression to perform well in both predictive accuracy and sparsity for the high-dimensional datasets [6]. Moreover, we first found that the multi-biomarker model had much better predictive capacity compared to the single serum biomarkers model. Also, we found that serum GLU had nonlinearity association with the risk of CVD, and serum TG and LDL-c had linearity association with the risk of CVD which provided relevant reference values to improve prevention of CVD.

Nevertheless, the limitation of our study was that it was a cross-sectional study, and the sample was limited, so we should perform additional verification of these data in other populations. Moreover, we should perform animal experiments in future studies.

## Conclusion

We found that serum FT4, TG and LDL-c were positively associated with the risk of CVD in single and multiple metabolites models, and the multi-biomarker model had much better predictive capacity compared to the single-biomarker of FT4. Moreover, serum TG and LDL-c had linearity relationship with the CVD incident.

## Supplementary information

**Additional file 1: Table S1.** The logistic regression analysis of multi-serum biomarkers and the risk of CVD which had no statistical association with CVD incident. **Table S2.** The unadjusted logistic regression analysis of single serum biomarker and the risk of CVD satisfied by gender. **Table S3.** The unadjusted logistic regression analysis of single serum biomarker and the risk of CVD satisfied by age. **Table S4.** Selected serum biomarkers associated with the risk of CVD. **Table S5.** The AUC of multi-biomarker model and single-biomarker model. **Fig. S1** Correlation map of serum biomarker levels among 321 patients. Only significant correlations are represented in the plot (*P* < 0.05, r = − 0.530 to 0.834), insignificant correlations were left blank. **Fig. S2** The restricted cubic spline for the association between serum biomarkers and the risk of CVD. The lines represent adjusted odds ratios based on restricted cubic splines for serum biomarker in the multiple-metals conditional regression model. Knots were placed at the 25th, 50th, and 75th percentiles of the serum biomarker distribution, and the reference value was set at the 50th percentile. Adjusted factors were age(≤ 60 and > 60 years old), the history of hypertension, diabetes, smoking and drinking. **Fig. S3** The elastic regression of multi-biomarker and CVD incident. **A**: The prediction error of the elastic regression model in function of the penalty parameter (log10 λ). **B:** The elastic solution path, with the coefficient profiles for serum metals as a function of the penalty parameter (log10 λ). Increasing values for λ, pose a more stringent penalty on the regression coefficients, shrinking more coefficients to zero. The horizontal red line depicts the cross-validated optimum of λ (minimum MSE), the dashed red line depicts the highest value of λ where the MSE was within one standard error (SE) of the minimum MSE. **Fig. S4** ROC curves of single biomarker and multi-biomarkers in different models. Multi-biomarker model: FT4, TG, GLU, HLD-c, LDL-c.

## Data Availability

The datasets used and/or analyzed during the current study are available from the corresponding author on reasonable request.

## Abbreviations

CVD: Cardiovascular disease
GLU: Glucose
CREA: Creatinine
HDL-c: High density lipoprotein cholesterol
LDL-c: Low density lipoprotein cholesterol
Hcy: Homocysteine
TSH: Thyroid stimulating hormone
