# Supplementary material for: Associations of multiple serum biomarkers and the risk of cardiovascular disease in China
Source: BMC Cardiovasc Disord. 2020 Sep 29;20:426. doi: 10.1186/s12872-020-01696-7 (PMC7523396; doi:10.1186/s12872-020-01696-7)
Supplement: Supplementary file 1 — Additional file 1: Table S1. The logistic regression analysis of multi-serum biomarkers and the risk of CVD which had no statistical association with CVD incident. Table S2. The unadjusted logistic regression analysis of single serum biomarker and the risk of CVD satisfied by gender. Table S3. The unadjusted logistic regression analysis of single serum biomarker and the risk of CVD satisfied by age. Table S4. Selected serum biomarkers associated with the risk of CVD. Table S5. The AUC of multi-biomarker model and single-biomarker model. Fig. S1 Correlation map of serum biomarker levels among 321 patients. Only significant correlations are represented in the plot (P < 0.05, r = − 0.530 to 0.834), insignificant correlations were left blank. Fig. S2 The restricted cubic spline for the association between serum biomarkers and the risk of CVD. The lines represent adjusted odds ratios based on restricted cubic splines for serum biomarker in the multiple-metals conditional regression model. Knots were placed at the 25th, 50th, and 75th percentiles of the serum biomarker distribution, and the reference value was set at the 50th percentile. Adjusted factors were age(≤ 60 and > 60 years old), the history of hypertension, diabetes, smoking and drinking. Fig. S3 The elastic regression of multi-biomarker and CVD incident. A: The prediction error of the elastic regression model in function of the penalty parameter (log10 λ). B: The elastic solution path, with the coefficient profiles for serum metals as a function of the penalty parameter (log10 λ). Increasing values for λ, pose a more stringent penalty on the regression coefficients, shrinking more coefficients to zero. The horizontal red line depicts the cross-validated optimum of λ (minimum MSE), the dashed red line depicts the highest value of λ where the MSE was within one standard error (SE) of the minimum MSE. Fig. S4 ROC curves of single biomarker and multi-biomarkers in different models. Multi-biomarker model: FT4, TG, G [file 12872_2020_1696_MOESM1_ESM.docx]

**Supplementary Tables**

Table S1 The logistic regression analysis of multi-serum biomarkers and the risk of CVD which had no statistical association with CVD incident

|  | n/(case/control) | Unadjusted OR (95%CI) | *P* | Adjusted  OR (95%CI) | *P* |
| --- | --- | --- | --- | --- | --- |
| TSH (mIU/L) |  |  |  |  |  |
| T1 (≤1.54) | 52/54 | 1.00 (Reference) |  | 1.00 (Reference) |  |
| T2(1.54-2.38) | 55/53 | 1.08 (0.63, 1.84) | 0.785 | 1.25 (0.70, 2.23) | 0.453 |
| T3 (>2.38) | 54/53 | 1.06 (0.62, 1.81) | 0.837 | 1.21 (0.67, 2.18) | 0.523 |
| *P* trend |  | 0.859 |  | 0.572 |  |
| FT3 (pmol/L) |  |  |  |  |  |
| T1 (≤4.54) | 42/55 | 1.00 (Reference) |  | 1.00 (Reference) |  |
| T2(4.54-5.17) | 54/52 | 1.36 (0.78, 2.37) | 0.276 | 1.21 (0.67, 2.20) | 0.527 |
| T3 (>5.17) | 65/53 | 1.61 (0.94, 2.76) | 0.086 | 1.77 (0.98, 3.20) | 0.058 |
| P trend |  | 0.094 |  | 0.053 |  |
| T3 (nmol/L) |  |  |  |  |  |
| T1(≤1.84) | 55/54 | 1.00 (Reference) |  | 1.00 (Reference) |  |
| T2(1.84-2.09) | 49/53 | 0.91 (0.53, 1.56) | 0.725 | 0.91 (0.51, 1.63) | 0.750 |
| T3(>2.09) | 57/53 | 1.06 (0.62, 1.79) | 0.841 | 1.05 (0.59, 1.85) | 0.869 |
| *P* trend |  | 0.830 |  | 0.858 |  |
| UA (μ mol/L) |  |  |  |  |  |
| T1 (≤229) | 38/55 | 1.00 (Reference) |  | 1.00 (Reference) |  |
| T2 (229-295) | 54/53 | 1.48 (0.84, 2.58) | 0.175 | 1.32 (0.72, 2.42) | 0.374 |
| T3 (>295) | 69/52 | 1.92 (1.11, 3.32) | 0.020 | 1.59 (0.85, 2.98) | 0.148 |
| *P* trend |  | 0.023 |  | 0.159 |  |
| TC (mmol/L) |  |  |  |  |  |
| T1 (≤4.4) | 50/55 | 1.00 (Reference) |  | 1.00 (Reference) |  |
| T2 (4.4-5.24) | 50/52 | 1.06 (0.61 1.83) | 0.840 | 1.20 (0.66, 2.16) | 0.550 |
| T3 (>5.24) | 61/53 | 1.27 (0.74 2.15) | 0.384 | 1.60 (0.88, 2.91) | 0.121 |
| *P* trend |  | 0.374 |  | 0.118 |  |
| HDL-c (mmol/L) |  |  |  |  |  |
| T1 (≤1.62) | 77/54 | 1.00 (Reference) |  | 1.00 (Reference) |  |
| T2 (1.62-1.27) | 47/53 | 0.62 (0.37, 1.05) | 0.076 | 0.61 (0.35, 1.07) | 0.086 |
| T3 (>1.27) | 37/53 | 0.49 (0.28, 0.85) | 0.010 | 0.57 (0.30, 1.06) | 0.076 |
| *P* trend |  | 0.010 |  | 0.070 |  |
| HCY (μmol/L) |  |  |  |  |  |
| T1 (≤10.1) | 56/55 | 1.00 (Reference) |  | 1.00 (Reference) |  |
| T2 (10.1-12.9) | 57/52 | 1.08 (0.63, 1.83) | 0.785 | 0.92 (0.51, 1.65) | 0.779 |
| T3 (>12.9) | 48/53 | 0.89 (0.52, 1.53) | 0.670 | 0.63 (0.34, 1.17) | 0.145 |
| *P* trend |  | 0.622 |  | 0.128 |  |

Adjusted by age(≤ 60 and > 60 years old.), gender, the history of hypertension, diabetes, smoking and drinking.

Hcy, homocysteine; TC, total cholesterol; TG, triglyceride; HDL-c, high-density lipoprotein cholesterol.

Table S2 The unadjusted logistic regression analysis of single serum biomarker and the risk of CVD satisfied by gender

| Biomarkers | Male | |  | Female | |
| --- | --- | --- | --- | --- | --- |
|  | Unadjusted OR (95%CI) | *P* |  | Unadjusted OR (95%CI) | *P* |
| **FT4** |  |  |  |  |  |
| T1 (≤15.77) | 1.00 (Reference) |  |  | 1.00 (Reference) |  |
| T2(15.77-18.34) | 2.04 (0.87, 4.79) | 0.103 |  | 2.04 (0.87, 4.79) | 0.103 |
| T3(>18.34) | 2.1 (0.95, 4.65) | 0.067 |  | 2.1 (0.95, 4.65) | 0.067 |
| *P* trend | 0.083 |  |  | 0.235 |  |
| **T4** |  |  |  |  |  |
| T1 (≤91.06) | 1.00 (Reference) |  |  | 1.00 (Reference) |  |
| T2(91.06-113.2) | 2.74 (1.24, 6.06) | 0.013 |  | 1.14 (0.51, 2.56) | 0.757 |
| T3(>113.2) | 1.5 (0.69, 3.26) | 0.307 |  | 1.36 (0.60, 3.08) | 0.464 |
| *P* trend | 0.248 |  |  | 0.452 |  |
| **GLU** |  |  |  |  |  |
| T1 (≤4.86) | 1.00 (Reference) |  |  | 1.00 (Reference) |  |
| T2 (4.86-5.5) | 1.02 (0.42, 2.45) | 0.965 |  | 1.19 (0.52, 2.75) | 0.683 |
| T3 (>5.5) | 3.09 (1.42, 6.72) | 0.005 |  | 2.4 0(1.10, 5.24) | 0.028 |
| *P* trend | 0.001 |  |  | 0.016 |  |
| **CREA** |  |  |  |  |  |
| T1 (≤56) | 0.41 (0.14, 1.22) | 0.108 |  | 0.58 (0.16, 2.04) | 0.392 |
| T2 (56-66) | 0.6 (0.18, 1.99) | 0.406 |  | 0.68 (0.34, 1.34) | 0.262 |
| T3 (>66) | 1.00 (Reference) |  |  | 1.00 (Reference) |  |
| *P* trend | 0.076 |  |  | 0.208 |  |
| **TG** |  |  |  |  |  |
| T1 (≤1.00) | 1.00 (Reference) |  |  | 1.00 (Reference) |  |
| T2 (1-1.62) | 1.29 (0.57, 2.94) | 0.537 |  | 2.16 (0.89, 5.26) | 0.09 |
| T3 (>1.62) | 2.14 (0.98, 4.69) | 0.057 |  | 3.70 (1.57, 8.72) | 0.003 |
| *P* trend | 0.05 |  |  | 0.003 |  |
| **LDL-c** |  |  |  |  |  |
| T1 (≤2.44) | 1.00 (Reference) |  |  | 1.00 (Reference) |  |
| T2 (2.44-3.02) | 1.33 (0.62, 2.87) | 0.462 |  | 1.59 (0.68, 3.71) | 0.284 |
| T3 (>3.02) | 2.43 (1.07, 5.54) | 0.035 |  | 1.9 (0.88, 4.11) | 0.101 |
| *P* trend | 0.035 |  |  | 0.108 |  |

Table S3 The unadjusted logistic regression analysis of single serum biomarker and the risk of CVD satisfied by age

| Biomarkers | ≤63 years old | |  | ＞63 years old | | |
| --- | --- | --- | --- | --- | --- | --- |
|  | Unadjusted | *P* |  | Unadjusted | *P* | |
|  | OR (95%CI) |  |  | OR (95%CI) |  | |
| **FT4** |  |  |  |  |  |  |
| T1 (≤15.77) | 1.00 (Reference) |  |  | 1.00 (Reference) |  |  |
| T2(15.77-18.34) | 1.848 (0.876, 3.899) | 0.107 |  | 1.789 (0.721, 4.444) | 0.21 |  |
| T3(>18.34) | 2.167 (1.031, 4.555) | 0.041 |  | 1.571 (0.638, 3.868) | 0.325 |  |
| *P* trend | 0.044 |  |  | 0.401 |  |  |
| **T4** |  |  |  |  |  |  |
| T1 (≤91.06) | 1.00 (Reference) |  |  | 1.00 (Reference) |  |  |
| T2(91.06-113.2) | 1.738 (0.835, 3.62) | 0.14 |  | 1.449 (0.628, 3.347) | 0.385 |  |
| T3(>113.2) | 1.292 (0.608, 2.749) | 0.505 |  | 1.483 (0.636, 3.46) | 0.362 |  |
| *P* trend | 0.557 |  |  | 0.365 |  |  |
| **GLU** |  |  |  |  |  |  |
| T1 (≤4.86) | 1.00 (Reference) |  |  | 1.00 (Reference) |  |  |
| T2 (4.86-5.5) | 0.938 (0.416, 2.116) | 0.878 |  | 1.176 (0.463, 2.99) | 0.733 |  |
| T23 (>5.5) | 3.065 (1.486, 6.319) | 0.002 |  | 2.143 (0.917, 5.007) | 0.078 |  |
| *P* trend | <0.001 |  |  | 0.054 |  |  |
| **CREA** |  |  |  |  |  |  |
| T1 (≤56) | 0.768 (0.368, 1.605) | 0.483 |  | 1.173 (0.492, 2.795) | 0.719 |  |
| T2 (56-66) | 0.991 (0.492, 1.996) | 0.98 |  | 1.005 (0.447, 2.259) | 0.99 |  |
| T3 (>66) | 1.00 (Reference) |  |  | 1.00 (Reference) |  |  |
| *P* trend | 0.978 |  |  | 0.988 |  |  |
| **TG** |  |  |  |  |  |  |
| T1 (≤1.00) | 1.00 (Reference) |  |  | 1.00 (Reference) |  |  |
| T2 (1-1.62) | 1.846 (0.809, 4.215) | 0.146 |  | 1.316 (0.557, 3.11) | 0.532 |  |
| T3 (>1.62) | 2.969 (1.365, 6.457) | 0.006 |  | 2.667 (1.121, 6.345) | 0.027 |  |
| *P* trend | 0.006 |  |  | 0.021 |  |  |
| **LDL-c** |  |  |  |  |  |  |
| T1 (≤2.44) | 1.00 (Reference) |  |  | 1.00 (Reference) |  |  |
| T2 (2.44-3.02) | 1.091 (0.52, 2.287) | 0.818 |  | 2.196 (0.892, 5.405) | 0.087 |  |
| T3 (>3.02) | 1.636 (0.799, 3.352) | 0.178 |  | 2.176 (0.935, 5.067) | 0.071 |  |
| *P* trend | 0.169 |  |  | 0.09 |  |  |

Table S4 Selected serum biomarkers associated with the risk of CVD

| Biomarkers | Coefficients | Biomarkers | Coefficients |
| --- | --- | --- | --- |
| FT4 (pmol/L) | 0.019 | HDL-c(mmol/L) | -0.224 |
| GLU (mmol/L) | 0.112 | LDL-c(mmol/L) | 0.177 |
| TG (mmol/L) | 0.063 |  |  |
| Selected Biomarkers | 5 |  |  |

Biomarkers was continuous various in the elastic net regression.

Table S5 The AUC of multi-biomarker model and single-biomarker model

| Model | AUC （95%CI） | *P** |
| --- | --- | --- |
| Multi-biomarker model | 0.660 (0.601, 0.720) |  |
| FT4 *vs* Multi- biomarker model | 0.569 (0.506, 0.632) | 0.038 |
| GLU *vs* Multi- biomarker model | 0.636 (0.575, 0.697) | 0.578 |
| TG *vs* Multi- biomarker model | 0.628 (0.568, 0.689) | 0.458 |
| LDL-c *vs* Multi- biomarker model | 0.580 (0.518, 0.642) | 0.068 |

* Z test.

**Supplementary Figures**

**
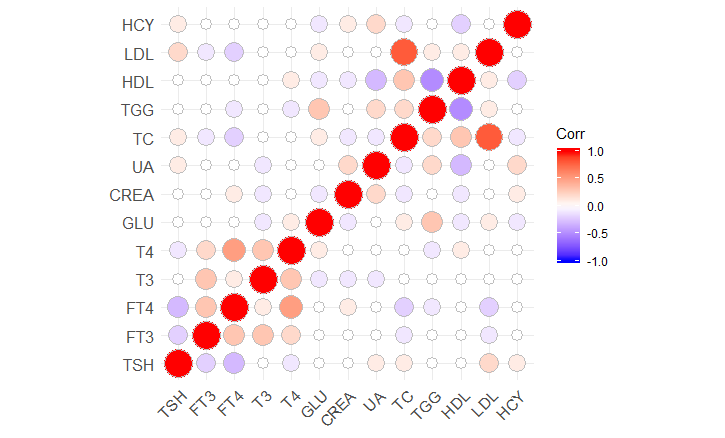
** **Figure S1** Correlation map of serum biomarker levels among 321 patients. Only significant correlations are represented in the plot (*P* < 0.05, r = −0.530 to 0.834), insignificant correlations were left blank.


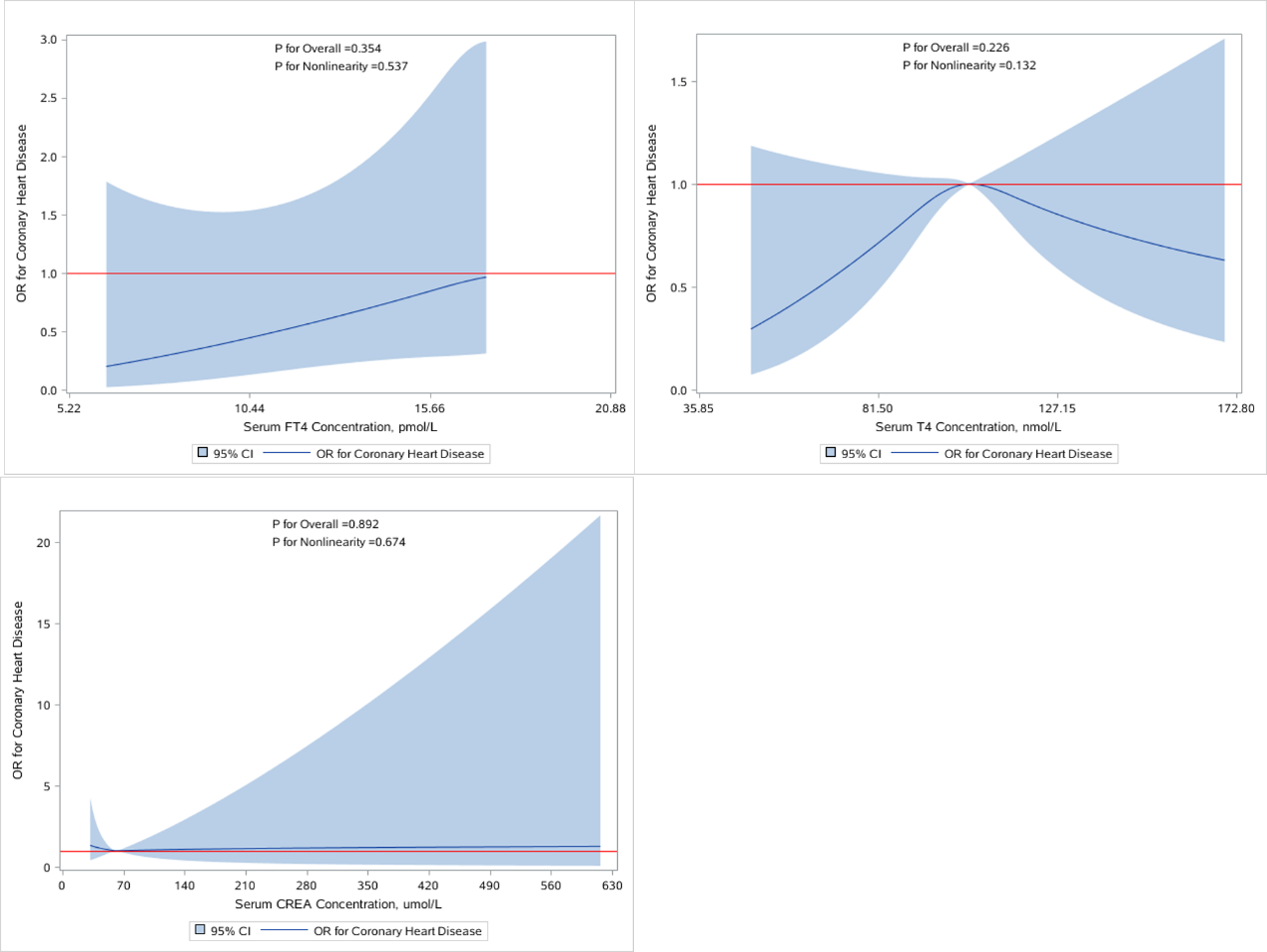


**Figure S2** The restricted cubic spline for the association between serum biomarkers and the risk of CVD. The lines represent adjusted odds ratios based on restricted cubic splines for serum biomarker in the multiple-metals conditional regression model. Knots were placed at the 25th, 50th, and 75th percentiles of the serum biomarker distribution, and the reference value was set at the 50th percentile. Adjusted factors were age(≤ 60 and ＞ 60 years old), the history of hypertension, diabetes, smoking and drinking.


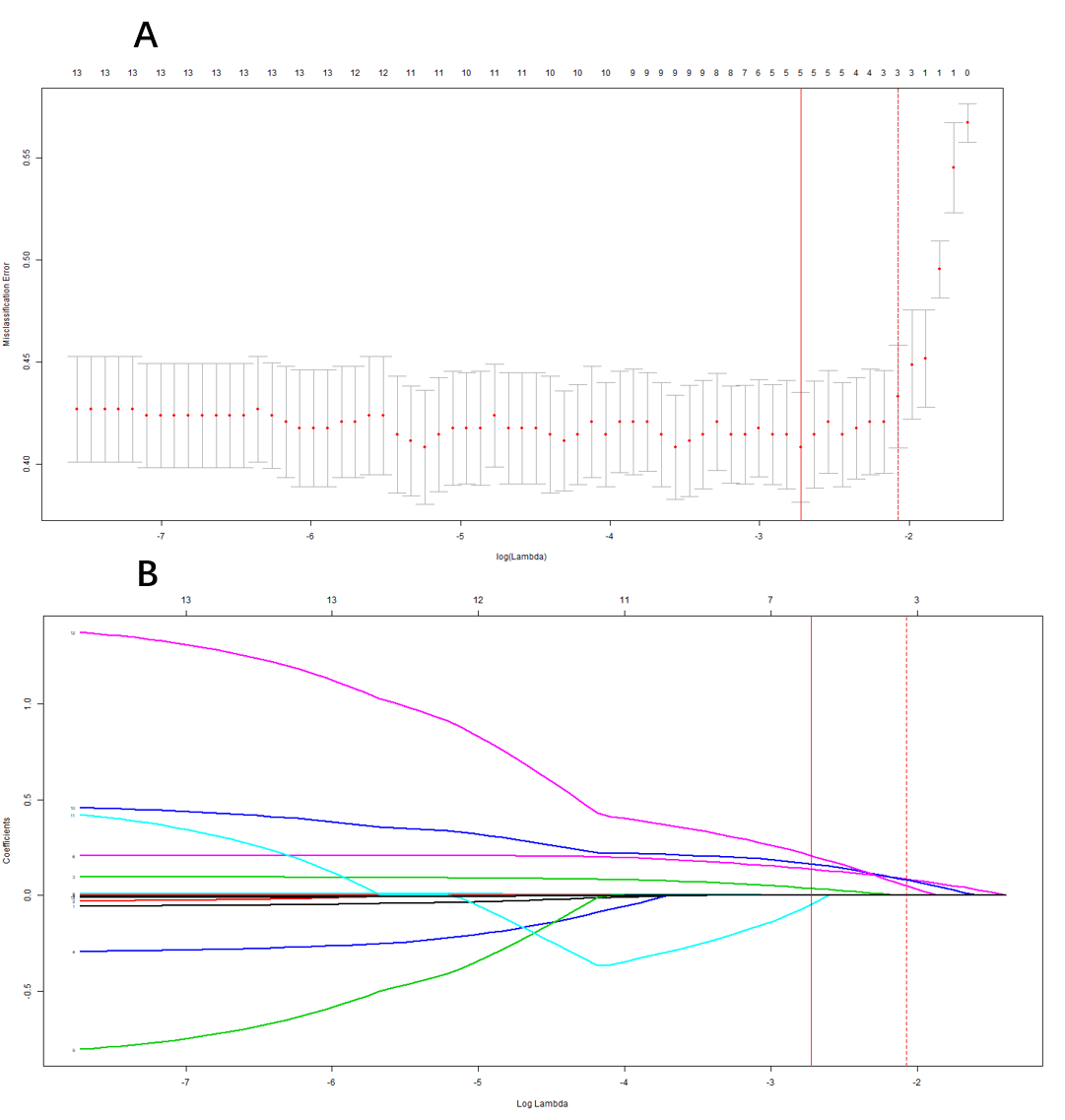


**Figure S3** The elastic regression of multi-biomarker and CVD incident. **A**: The prediction error of the elastic regression model in function of the penalty parameter (log10 λ). **B:** The elastic solution path, with the coefficient profiles for serum metals as a function of the penalty parameter (log10 λ). Increasing values for λ, pose a more stringent penalty on the regression coefficients, shrinking more coefficients to zero. The horizontal red line depicts the cross-validated optimum of λ (minimum MSE), the dashed red line depicts the highest value of λ where the MSE was within one standard error (SE) of the minimum MSE.

**Figure S4**


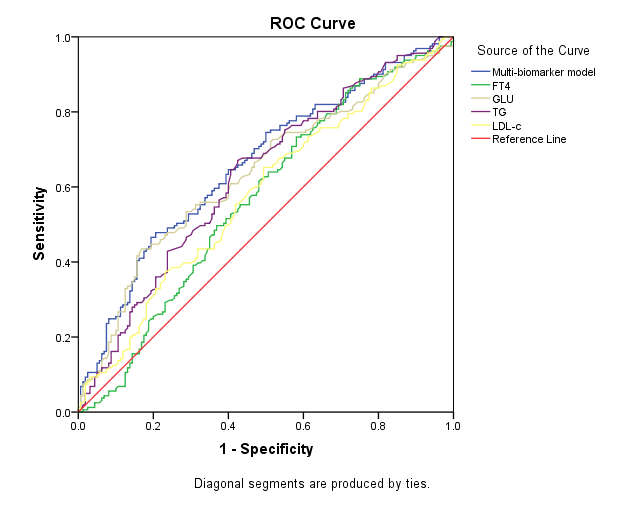


**Figure S4** ROC curves of single biomarker and multi-biomarkers in different models. Multi-biomarker model: FT4, TG, GLU, HLD-c, LDL-c.
